# Self-Efficacy as a Positive Youth Development Construct: A Conceptual Review

**DOI:** 10.1100/2012/452327

**Published:** 2012-04-29

**Authors:** Sandra K. M. Tsang, Eadaoin K. P. Hui, Bella C. M. Law

**Affiliations:** ^1^Department of Social Work and Social Administration, Faculty of Social Sciences, The University of Hong Kong, Pokfulam Road, Hong Kong; ^2^Division of Learning Development and Diversity, Faculty of Education, The University of Hong Kong, Pokfulam Road, Hong Kong

## Abstract

Self-efficacy denotes people's beliefs about their ability to perform in different situations. It functions as a multilevel and multifaceted set of beliefs that influence how people feel, think, motivate themselves, and behave during various tasks. Self-efficacy beliefs are informed by enactive attainment, vicarious experience, imaginal experiences, and social persuasion as well as physical and emotional states. These beliefs are mediated by cognitive, motivational, affective, and selection processes to generate actual performance. Self-efficacy development is closely intertwined with a person's experiences, competencies, and developmental tasks in different domains at different stages in life. This paper reviews the literature to outline the definition and theoretical conceptualizations of the construct originally devised by Bandura that have flourished since the 1990s. Drawing from the studies of the construct to assess self-efficacy, and to inform positive youth development, the paper will present the determinants of the development of self-efficacy beliefs and identify the connection between self-efficacy and adolescent developmental outcomes. The paper will conclude with strategies to enhance youth self-efficacy and proposals for future research directions.

## 1. Background

Since the 1970s, the social cognitive theory proposed by Bandura [1–3] has been one of the most influential theories used to guide the understanding of human behavior and the motivational determinants of such behavior. The theory advocates a theme of “triadic reciprocity” which asserts that a person's behavior is constantly under the reciprocal influence of the environment and personal cognitions. When applied in the context of adolescent development, such as academic performance, this theory suggests that an adolescent's academic performance (behavior) is influenced by how this adolescent's beliefs (cognitions) are affected by the support provided by his or her significant others, including parents, teachers, and peers (the environment). Bandura argues that self-efficacy is the most pivotal factor affecting a person's cognition, and his assertion has popularized self-efficacy studies since the 1990s.

The following sections aim to present findings and observations from a review of the literature on the definition, assessment, theoretical conceptualizations, adolescent development outcomes, and promotion strategies of self-efficacy, with specific reference to positive youth development. Identified research gaps and suggestions for future research will also be presented.

## 2. Definition of Self-Efficacy

Self-efficacy refers to one's beliefs in one's capability to organize and execute the courses of action required to achieve given results [4]. In the 1994 Encyclopedia of Human Behavior [5], Bandura emphasized that “self-efficacy beliefs determine how people feel, think, motivate themselves and behave” (p.71). The concept has been used in research in two different ways: as “task self-efficacy” denoting the perceived ability to perform a particular behavior and as “coping self-efficacy” denoting the perceived ability to prevent, control, or cope with potential difficulties that might be encountered when engaged in a particular performance [6, 7]. In the context of seeking evidence-informed ways to promote positive youth development, these two perspectives are both very useful because adolescents enjoy optimal physical growth and energy and are open to the formulation of their self-identity [8]. They actively address the potentials and possibilities as well as the challenges and crises of their adolescent developmental stage [9]. Their beliefs in their self-efficacy for different tasks and the cumulative effects of such beliefs will significantly influence their immediate and long-term development.

Self-efficacy is experimentally validated through substantial causality-testing research projects involving “different modes of efficacy induction, diverse populations, using both inter-individual and intra-individual verification, in all sorts of domains of functioning, and with micro level and macro level relations” (Bandura, 1997, as cited in p.18 [10]). Results suggest that self-efficacy functions as a multilevel and multifaceted set of beliefs, each differing in level, strength, and generativity [11].

That means, aside from a general perception of self-efficacy, there can be very specific beliefs in self-efficacy regarding different domains of oneself (e.g., physical strength in soccer, or the stamina to prepare for a difficult mathematics test). Self-efficacy beliefs also vary in level, strength, and generativity across different domains.

Using language self-efficacy for an illustration, the self-efficacy level refers to variations of self-efficacy beliefs across the mastery of a first and second language; the strength of perceived self-efficacy is indicated by the degree of certainty in using the language in social or formal occasions, while generativity refers to the transfer of self-efficacy beliefs across different language assignments (e.g., written or oral presentations). Each belief and its impact are sensitive to variations in situation, context, and task, and they orchestrate and steer a person's course of actions (performance) that generate outcomes in the form of positive or negative physical, social, and self-evaluation effects [4].

## 3. Assessment of Self-Efficacy

Self-efficacy assessment is needed for understanding the nature and strength of beliefs that influence performance. Quantitative and qualitative assessment measures and strategies have been devised to assess general self-efficacy, as well as sources and processes of self-efficacy. Self-efficacy is best assessed within the consideration of contextual factors in order to discern whether it plays a mediating, moderating, or other role in a behavioral performance. In the case of secondary school students' development, contextual factors like gender, ethnicity, academic ability, and academic domain should be priority concerns.

Usher and Pajares [12] described and critically reviewed both quantitative and qualitative means to assess sources of self-efficacy in school. They found that scales using Likert-type items have been created to assess sources like mastery experience, vicarious experience, social persuasions, and physiological state. These sources have varied psychometric properties when tested with construct or explanatory factor analysis, or construct validity and internal reliability. However, they also found that the reliability measures on vicarious experiences have consistently been notably low, and more studies are needed to strengthen such measures.

Usher and Pajares [12] also identified some qualitative methods that can be used to assess self-efficacy and sources of self-efficacy under different personal, social, situational, and temporal conditions. Methods include grounded theory, ethnography, classroom observations, case studies, interviews, self-reports on recalled reasons for self-efficacy judgments, and self-assigned weights of self-efficacy regarding academic performance. It was found that the semistructured interview is most useful for capturing both the objective and subjective aspects of self-efficacy beliefs, and the nature and processes of the influence of these beliefs on performance.

Qualitative methods are particularly useful for studying cases where individuals still harbor disabling self-doubts even though they have been recognized to have more than adequate competence in performing the task in question. Thus, it is important to synthesize the assessment of such sources with an assessment of psychological processes like motivation, emotion management, strategies in task selection, and problem-solving resourcefulness.

In view of the fact that self-efficacy is complex and context specific, there is a need for researchers to develop thorough measures that effectively assess the multidimensionality of the hypothesized sources and processes of self-efficacy, together with the strengths and dynamic interactions of these sources and processes. O'Sullivan and Strauser [13] once stated that “It should be noted that for almost any behavior that can be imagined an efficacy scale has been developed” (p.257) (e.g., diabetes management efficacy scale, science-teacher efficacy scale, internet use efficacy scale, etc.).

While it appears that great advancement has been achieved in the assessment of self-efficacy, it has to be noted that generating some very task-specific assessment measures in the changing world where young people learn and live can be a time-consuming and even endless pursuit. It seems that while striking the right balance between generality and specificity, future research should still try to find the core elements of self-efficacy beliefs that are sensitive to intervention and that can be reliably and validly measured and compared for changes.

In Hong Kong, attempts to develop a self-efficacy scale for Chinese junior secondary school students have been made [14], and the psychometric properties of that scale are satisfactory. The scale consists of 7 items including statements like “When I face life difficulties, I feel helpless” that are to be answered in a 6-point Likert format. It is still a rather general self-efficacy scale for youths, but it is a big step forward in devising ways to measure culture-specific self-efficacy in young people in China. This is important as China is having an increasing influence on the world both in terms of the size of its population and its resource potentials. There is also evidence showing that because Chinese parents and children still value academic achievement as the most important facilitator for upward social mobility, they assert so much concern on academic performance that often high academic achievers still suffer from low academic self-efficacy [15–17].

All these things suggest that for Chinese, in addition to a general self-efficacy scale, other scales focusing on more specific domains like academic, social, sports, moral, information technology management, and social services also need to be developed in order to fully address the different aspects of youth talents and performance and to yield information on possible means of intervention.

## 4. Theories on Self-Efficacy

Research-informed theoretical formulations of self-efficacy drew from learning, cognitive, and social cognitive theories and were able to shed light on the nature, sources, and psychological processes involved in the formation of self-efficacy beliefs. Learning theories attempting to explain the emergence of behavior first focused on conditioning, and then on the consequences of behavior. Cognitive theories of learning introduced cognition into the behavior generation process and emphasized the consideration of gains or losses resulting from performing the said behavior as significant deciding factors. According to Klassen and Usher [18], “Bandura's Social Cognition Theory marks human functioning as the product of a dynamic interplay of personal, behavioral and environmental influences. These factors exert their influence through a process of reciprocal determinism, by which (a) personal factors in the form of cognition, affect, and biological events, (b) behavior, and (c) environmental influences interact” (p.3).

Research along this line shows that people's self-efficacy beliefs about their capabilities and about the outcomes of their efforts are particularly predictive of actual behavior, like academic performance and even vocational choices. Self-efficacy is also “associated with key motivational constructs like causal attributions, self-concept, optimism, achievement goal orientation, academic help-seeking, anxiety, and value” (p.751) [12] and is thus the most important construct of the social cognitive theory.

 The theory asserts that self-efficacy beliefs work through the four major psychological processes listed below to produce actual performance.

1. Cognitive processes: these include self-appraisal of capabilities, skills, and resources; goal selection; construction of success and failure scenarios in the goal accomplishment processes; generation and selection of problem-solving options; sustaining the necessary attention and functioning for task completion.
2. Motivational processes: self-efficacy beliefs affect one's self-regulation of motivation. Three cognitive motivators, namely, “attribution,” “value of expected outcomes,” and “clarity and value of goals” have been identified as being influenced by self-efficacy beliefs.
3. Affective processes: a person's self-perception of coping abilities affects the person's arousal threshold and their tolerance of emotional threats like anxiety and depression [11]. Even the process and outcome of threat management can be affected by procedures like guiding imagery to adjust anxiety symptoms when encountering stressors [19].
4. Selection processes: decisions on choice of residence, career, family setup, and even use of time can directly influence a person's functioning. In order to attain the outcomes they are interested in, people with high self-efficacy are more proactive in selecting and creating a physical and social environment that matches their perceived capabilities and resources. Their chances of successful goal attainment and personal development are also maximized in the process.

According to Bandura [4] and Maddux and Gosselin [7], self-efficacy beliefs formed through the above processes are not static. They are constantly informed, energized, or depleted through at least five identifiable primary sources that are affected by a person's interpretations of former and current experiences.

1. Mastery experiences: cognitive processes working on the previous experience of mastery or success in an actual task performance will raise self-efficacy. Successful perseverance through some hardship in the task completion process can even reinforce the durability of self-efficacy. That explains why the adventure-based type of experiential training is both welcomed by young people and found to have a positive impact on their growth and development.
2. Vicarious experience: observation of successful task performance by social models (like parents and teachers), and by those whose capabilities are similar to oneself (like peers for young people), generates a strong sense of self-efficacy. Effective mastery and coping models, such as parents, teachers, or peers who cope competently with challenges, can demonstrate and stimulate the learning of skills and strategies [20]. These models can also promote the readiness of young people to put ideas into action, thus creating more chances for success that will further enhance self-efficacy.
3. Social persuasion: convincing verbal persuasion given by significant others, like parents and teachers [21, 22], can enhance a young person's self-efficacy, provided that the youth really possesses the capabilities in question. Failure to complete a task that was based on false expectations can do more to damage self-efficacy beliefs than to build them up. Successful social persuasion should include manipulation of all variables in the triadic reciprocity process: expansion of the behavior repertoire through skills training and environmental control to facilitate successful performance, as well as convincing persuasion of the desirability of the outcome. In recent years, there has been an emerging trend to introduce mature and successful adults from the community to serve as mentors for young people in order to expand the social capital of young people beyond family and school boundaries. The role modeling and guidance of these mentors should provide useful self-efficacy sources for young people.
4. Physiological and affective states: actual and perceived physiological and emotional conditions work directly through the affective processes described in the above section to influence a person's self-efficacy beliefs. These physiological and emotional conditions include physical and mental readiness for action, vulnerability to fatigue, and susceptibility to a decision to continue or give up. These states also influence the person's subscription to different ways of interpreting and handling all this information. These are particularly important for young people because young people possess important developmental resources like physical energy and emotional accessibility and can benefit greatly if such sources are optimized in time.
5. Imaginal experiences: imaginal rehearsal of successful or unsuccessful performance, be it deliberate or while ruminating, can improve coping strategy and enhance self-efficacy [7]. Examples include imagination-based interventions such as systematic desensitization and covert modeling [23]. In promoting youth self-efficacy, the use of experiential exercises and role playing in skills practice has been found to be helpful in expanding youth experience and preparation [24].

Careful understanding and manipulation of the above psychological processes and sources that influence the formation and functioning of self-efficacy beliefs should create promising avenues for the promotion of self-efficacy. In the context of positive youth development, Usher and Pajares [12] critically reviewed the literature on the sources of self-efficacy in school and proposed directions for research and enhancement strategies.

Suggestions include (a) paying attention to both a quantitative and a qualitative assessment of self-efficacy in order to fine-tune the theory and the conceptualization of the nature and the function of its sources and processes; (b) making self-efficacy considerations more context, task, age, gender, academic domain, academic level, and culture sensitive, while also examining their generalizability; (c) utilizing the relationship between the sources of self-efficacy to introduce even more creative enhancement strategies; (d) identifying if there are other sources of self-efficacy in addition to the four proposed by Bandura.

Specifically, Usher and Pajares identified an invitational approach [25] that suggests that the beliefs people develop about themselves and about others jointly form the perceptual lenses through which people view the world and appreciate new experiences. The messages (or invitations) that people receive and send are pivotal in creating self-efficacy beliefs. Bandura also stated that the interplay amongst the self-efficacy sources can be additive, relative, multiplicative, or configurative.

While Bandura nearly exclusively emphasizes the causal importance of self-efficacy beliefs in influencing final behavior, there is also increasing evidence drawing due attention to the importance of outcome expectancies in producing behavior. Some of the recent applications on young people include expectancy studies on indulgent behavior, like gambling, smoking [26], and cyber addiction [27]. There should also be more discussion on how to manage possible mismatches between self-efficacy and the knowledge and skills necessary for task performance, and how to help youths with low competence and inadequate work attitudes but high self-efficacy buildup functional competence and attitude. More studies are still needed to establish the specific role of each self-efficacy source and process and the role they play in informing and enhancing actual performance.

In recent years, self-efficacy studies have been giving more attention to the environmental variable, and to discussing individual versus collective self-efficacy. In a context like secondary schools where adolescents are constantly in close interaction with their peers and teachers, research should go beyond individual efficacy studies and examine the collective efficacy of the whole class, subgroups in the class, teachers and students as subgroups in a school, or one school versus others in open competitions with other schools [18].

As adolescents are still mainly under the influence of families and schools in their development, attempts to theorize and enhance adolescent development and performance should also give more attention to the efficacy beliefs of parents and teachers. The quality of the role performance of parents and teachers should be examined together with the impact of such on the development of young people's study habits, values and attitudes, health and social habits, and how they can avoid risky behavior.

## 5. Self-Efficacy and Adolescent Developmental Outcomes

Pajares [28] reviewed over 20 years of self-efficacy research and identified two main lines of study: (a) connecting self-efficacy beliefs with college major and vocational choices and (b) surveying the connections amongst self-efficacy, other psychological constructs, and academic performance. There are numerous research studies showing that self-efficacy beliefs help determine both task performance (whether people choose to attempt certain tasks, how they attempt the tasks) and coping (how people tackle challenges arising from trying to complete the task, the degree of anxiety and frustration they experience in the process). In the case of adolescents, Pajares and Urdan [29] showed that self-efficacy predicts academic areas and levels, while Brown and Lent [30] identified that self-efficacy predicts students' college major and career choices. In their 2008 review of the literature since 1977 on the sources of self-efficacy in school, Usher and Pajares [12] observed that self-efficacy is “associated with key motivational constructs such as causal attributions, self-concept, optimism, achievement goal orientation, academic help-seeking, anxiety, and value” (p.751). Self-efficacy is also connected to self-regulated learning, including students' decision to stay in school [31], and academic procrastination [32]. 

Aside from academic performance and study style, self-efficacy also has an impact on adolescents' performance in extracurricular activities like soccer [19]. A review of two school intervention projects aiming to promote students' self-efficacy and school mental health in Germany found that individualized task demands and specific teacher feedback enhance student self-efficacy, while social self-efficacy is fostered through a positive class climate with mutual support amongst students, and when teachers are sensitive to the individual needs of the students [24]. The students who finished the projects reported improved motivational orientations, coping with stress, and conflict solving. Cicognani [33] studied 342 adolescents and found that coping resources like self-efficacy helped them survive minor stressors and fostered psychological well-being and social support.

In recent years, research into the role of self-efficacy in the regulation of involvement in peer aggression and defending the victim [34], or in indulgent behavior like smoking [35], drinking [36], drug addiction [37], and internet usage [38] has also produced very promising results.

## 6. Promotion of Self-Efficacy in Adolescents

There is plenty of research evidence indicating that timely and strategic cultivation of positive self-efficacy in early adolescence is important and possible. In 1998, Richard Catalano and his colleagues in the University of Washington reviewed 25 effective “Positive Youth Development Programs in the United States” and found that each of these programs included a component to promote self-efficacy [39]. Popular themes included the enhancement of skills, responsibility, supportive relationships, and belonging. There is also an increased indication that the promotion strategies have to be age, gender, task, and culture specific to show the best results, and using self-efficacy evaluation measures tailored for the task to be mastered will also show the clearest intervention effect [18]. These findings have informed teaching in Hong Kong and research demonstrating their usefulness is just beginning to build up. Some of the strategies found useful for Chinese school children were competitions in vicarious learning for writing tasks [40], and delivering individual and formative evaluative feedback to foster self-efficacy in English vocabulary acquisition [41].

Aside from work done with individual adolescents, increasing attention is being paid to cultivate collective self-efficacy [18]. A whole class in a secondary school, or a group in a team project, or even a whole school, can also be used as a collective unit, depending on whether it is a class, group, or school-based task. Inclusion of the belief in efficacy, be it the team leader, a fellow student, or the responsible teacher or trainer, is also found to be useful in appreciating the full sources and dynamics of self-efficacy.

Since most children stay at home and then go to primary and secondary school for education before they enter tertiary education, parents and teachers should be important contextual agents to be included in studies of social cognitive theory. Surprisingly, a review of 244 articles on self-efficacy from the period 2000–2009 found that some 40% studied teachers while only 2% studied parents [18]. Fan and Williams [21] found that parental advising on study in English and family rules for watching television were positively linked to students' engagement and intrinsic motivation towards both English and mathematics. As most Chinese parents put a very high priority on supporting their children to achieve academically, and as home-school cooperation has been found to provide useful support for adolescent development [22], it is important that self-efficacy studies draw adequately from these two important contextual agents.

## 7. Research Gaps and Future Research Directions

Considering the current literature, and the review of self-efficacy studies from 1977 up to 2007 by Usher and Pajares [12] (Usher and Pajares used sources, antecedents, self-efficacy, and development in various combinations as search items), as well as the review by Klassen and Usher on 244 articles from 65 journals of self-efficacy studies [18], the following is recommended for future self-efficacy research, especially where adolescent positive development is concerned:

1. refine the measurement of the self-efficacy sources: each of the four named self-efficacy sources differs in nature, and they vary according to the task and the context in question, so that there should be source and task-specific assessments to detect any changes with adequate sensitivity;
2. foster new methods of inquiry: aside from purely quantitative measures, qualitative and mixed method assessment should also be used. In addition to self-administered questionnaires, interviews, and self-reported recall tasks, innovative research design should also be developed to capture the full interplay amongst the person, their behavior, and the environment in human functioning;
3. consider new elements and paths in social cognition theory: this might include new sources of self-efficacy like the invitational approach [25], optimism, and positive psychology, as well as the role of outcome expectancy [42]. There should be more investigation into the transformative experience in the formation of self-efficacy. Exploration into the neurobiological basis of self-efficacy, in adolescence and across the human life-span, should also be another productive agenda;
4. attend to collective efficacy: Klassen and Usher [18] found that during the period 2000–2009, education-related studies on collective efficacy were few and focused on teachers rather than students. It is high time such collective beliefs were better understood, and that individual and collective efficacies were put into proper perspective;
5. attend to gender, age, and cultural variations: according to a ten-year review [18], over 60% of the 244 articles reviewed were on N. America, with only 20% on Asia. With the growing impact of globalization, and communication without borders on the internet, more attention should be paid to different forms of culture when trying to understand the nature and dynamics of self-efficacy. Aside from describing the effects of gender, age, and cultural differences on self-efficacy, it is also important to find out the causes for such differences. As an example, the roles of an individual's gender orientation and personal style, as well as the role of the home, culture, school, and the mass media, should all be clearly discerned to sharpen the effectiveness of interventions. With a growing number of children with special educational needs, and the effect the complex interplay of challenges to their learning has on their self-efficacy and performance, due attention must be paid to understanding how to support such children, parents, and teachers in the best way.
